# Systematic analysis of TruSeq, SMARTer and SMARTer Ultra-Low RNA-seq kits for standard, low and ultra-low quantity samples

**DOI:** 10.1038/s41598-019-43983-0

**Published:** 2019-05-17

**Authors:** Marie-Ange Palomares, Cyril Dalmasso, Eric Bonnet, Céline Derbois, Solène Brohard-Julien, Christophe Ambroise, Christophe Battail, Jean-François Deleuze, Robert Olaso

**Affiliations:** 1 Centre National de Recherche en Génomique Humaine (CNRGH), Institut de Biologie François Jacob, CEA, 91057 Evry, France; 2 Université Paris-Saclay, 91190 Saint-Aubin, France; 3 Laboratoire de Mathématiques et Modélisation ďÉvry (LaMME), Université ďEvry Val ďEssonne, 91000 Evry, France; 4 UMR CNRS 8071, 91000 Evry, France; 5 Ecole Nationale Supérieure ďInformatique pour l’Industrie et l’Entreprise, ENSIIE, 91000 Evry, France; 6 USC INRA, 91000 Evry, France

**Keywords:** Transcriptomics, Transcriptomics, Data acquisition, Data acquisition

## Abstract

High-throughput RNA-sequencing has become the gold standard method for whole-transcriptome gene expression analysis, and is widely used in numerous applications to study cell and tissue transcriptomes. It is also being increasingly used in a number of clinical applications, including expression profiling for diagnostics and alternative transcript detection. However, despite its many advantages, RNA sequencing can be challenging in some situations, for instance in cases of low input amounts or degraded RNA samples. Several protocols have been proposed to overcome these challenges, and many are available as commercial kits. In this study, we systematically test three recent commercial technologies for RNA-seq library preparation (TruSeq, SMARTer and SMARTer Ultra-Low) on human biological reference materials, using standard (1 mg), low (100 ng and 10 ng) and ultra-low (<1 ng) input amounts, and for mRNA and total RNA, stranded and unstranded. The results are analyzed using read quality and alignment metrics, gene detection and differential gene expression metrics. Overall, we show that the TruSeq kit performs well with an input amount of 100 ng, while the SMARTer kit shows decreased performance for inputs of 100 and 10 ng, and the SMARTer Ultra-Low kit performs relatively well for input amounts <1 ng. All the results are discussed in detail, and we provide guidelines for biologists for the selection of an RNA-seq library preparation kit.

## Introduction

RNA molecules play an essential role in numerous biological processes. With the recent evolution of next-generation sequencing technologies, investigation of the wide diversity of RNA species using high-throughput RNA sequencing (RNA-seq) is now easier than ever before. Unlike older techniques such as quantitative reverse transcription and microarrays, RNA-seq is an open platform in the sense that it does not depend on genome annotation or predefined and species-specific probes for transcript measurement, thus allowing the detection of both known and novel transcripts, including variants and rare transcripts^1^. RNA-seq has been shown to have a greater dynamic range than microarrays, increasing the potential for the detection of variation within samples^2^. This technology can identify tens of thousands of differentially expressed genes and their isoforms. It can also detect mutations and germline variations for hundreds of thousands of expressed genetic variants, and can detect gene fusions and splice variants^3,4^. In addition, RNA-seq can identify various classes of non-coding RNAs, such as microRNAs, PIWI-interacting RNAs and tRNAs^4^. RNA-seq techniques are being increasingly used in clinical applications. For instance, the recent breast cancer guidelines support the usage of mRNA-based prognostic assays to assist in treatment decisions alongside other clinico-pathological factors^5^. These assays could also provide a better view of alternative transcript variants which arise from splicing alterations or structural variants and are implicated in a range of human diseases such as developmental disorders^6^, neurodegenerative disorders^7^ and cancers^8^. Thus, RNA-seq will most likely transition in the very near future from a discovery tool to a diagnostic tool with clinical applications such as patient stratification, diagnosis and personalized treatment^1^. Producing high quality RNA-seq libraries and sequencing results can be challenging due to the multiple factors and conditions involved. For instance, in many cases it is necessary to reduce the representation of abundant ribosomal RNAs (rRNA) in libraries prior to sequencing. Sometimes libraries have to be prepared from poor quality samples such as formalin-fixed, paraffin-embedded (FFPE) samples. In other cases, working with sorted cell subtypes, minute tissue samples or even single cells can result in low or ultra low sample input amounts. To overcome these challenges, several methods and kits have been developed, each with its own strengths and weaknesses. The relative merits of each of these methods compared to methods which use a standard high quality and high quantity input should be determined by a careful comparison of multiple metrics^9–13^. Several studies have investigated the impact of degraded RNA input^14–18^, while others have focused on low RNA inputs^17,19–21^ and some studies have focused on both aspects^17,18^, or on the general properties of the RNA-seq protocols^3^. Here, in the light of recent developments and progress in RNA-seq library protocols, we use human input samples to evaluate three different recently developed commercial kits used for RNA-seq library preparation: TruSeq (Illumina), SMARTer (Clontech/Takara Bio) and SMARTer Ultra-Low (Clontech/Takara Bio). Furthermore, we test these kits for unstranded and stranded conditions, mRNA and total RNA input selection and for three different quantities of input material: standard (1 *μ*g), low (100 ng and 10 ng) and ultra-low (<1 ng). In total, we sequence 80 different libraries representing the different preparation kits/input material combinations using high-throughput sequencing. The results are then analyzed using read quality and alignment metrics, gene detection and differential gene expression metrics. These particular RNA sequencing kits were chosen for three reasons: (1) they are relatively new to the market and are in common use, especially in medium to large sequencing centers, (2) some of the kits are optimized for automated library preparation and automation capability is obviously an important factor to consider when processing large numbers of samples, and (3), they are specifically adapted to low and ultra-low input amounts, an important consideration since more and more projects today have limited RNA inputs (for example when tissue or condition-specific cells are sorted by FACS before sequencing). Other commercial kits could have been included in this study, for example, the NEBNext Ultra II kit (New England Biolabs), the NEBNext Single Cell/Low Input RNA Library Prep Kit (New England Biolabs) and the Ovation RNA-seq V2 system (NUGEN). However, our choice was restricted by budget and time constraints. No previous study, to our knowledge, has combined the particular kits selected here with the input amounts, sampling levels and all the different metrics included in this analysis. Finally, based on the data generated and the gene detection and differential expression analysis, we discuss below the performance of the different kits and input amounts, and their impact, in general, on the investigation of the transcriptomic landscape.

## Results

### RNA samples, sequencing protocols and study design

In order to evaluate the performance of RNA-seq methods for profiling standard, low and ultra-low quantity samples, we compared the quality metrics, gene detection and differential analysis capacities of three different groups of RNA-seq kits. The different conditions used are detailed in Table 1. The first group is based on the widely used Illumina TruSeq technology which we used for total RNA and mRNA extraction, unstranded and stranded, with input amounts of 1 *μ*g and 100 ng. The second group is based on SMARTer technology which we used for total RNA extraction, performing the ribosomal RNA depletion with two different kits (RiboGone and RiboZero) and using input amounts of 100 ng and 10 ng. The third group is based on a combination of SMARTer Ultra-Low plus Nextera technologies for ultra-low input amounts of 750 pg and 130 pg. For the RNA input material, we used two human RNA sample preparations previously used in reference RNA-seq studies^9,11–13^ (see methods). All samples were sequenced in duplicate giving a total of 80 sequenced libraries. Paired-end reads were then checked for quality, aligned to the human genome, and counts were generated for all human genes. Lastly, for the gene detection and differential analysis studies, mapped reads were sampled at four different levels. The sampling level reflects the sequencing depth at which the libraries will be sequenced. This important parameter can of course influence the global cost of the project, but also the type of downstream analysis that can be done. For instance, analyzing transcript isoforms would require a high sequencing depth, whereas restricting the analysis to the most highly expressed genes could be done safely at a much lower sequencing depth.

**Table 1:** Overview of the different RNA preparation kits and conditions analyzed in this study.

Kit	Manufacturer	RNA type	Input (ng)	Stranded	Acronym	PC
**Illumina technology**						
mRNA TruSeq	Illumina	mRNA	1000	no	mRNATruseq_1 ug	13
Stranded mRNA TruSeq	Illumina	mRNA	1000	yes	ssmRNATruseq_1 ug	13
Stranded total RNA TruSeq	Illumina	total RNA	1000	yes	ssTotRNATruseq_1 ug	14
Stranded mRNA TruSeq	Illumina	mRNA	100	yes	ssmRNATruseq_100 ng	15
Stranded total RNA TruSeq	Illumina	total RNA	100	yes	ssTotRNATruseq_100 ng	15
**SMARTer technology**						
RiboGone Stranded SMARTer	Takara Bio	total RNA	100	yes	RiboG_ssTotSmarter_100 ng	18
RiboZero Gold stranded SMARTer	Illumina + Takara Bio	total RNA	100	yes	RiboZ_ssTotSmarter_100 ng	18
RiboZero Gold stranded SMARTer	Illumina + Takara Bio	total RNA	10	yes	RiboZ_ssTotSmarter_10 ng	18
**SMARTer Ultra-Low technology**						
SMARTer UL + Nextera	Takara Bio + Illumina	mRNA	1 (0.75)	no	mRNASmarterUL_XT1 ng_750 pg	11/12
SMARTer UL + Nextera	Takara Bio + Illumina	mRNA	1 (0.13)	no	mRNASmarterUL_XT1 ng_130 pg	11/12

PC: number of PCR cycles done for each application. For the Ultra-Low SMARTer kits, the first digit is the number of cycles for the LD-PCR (first step), and the second digit is the number of cycles for the PCR (second step).

### Read quality metrics

**Figure 1:**
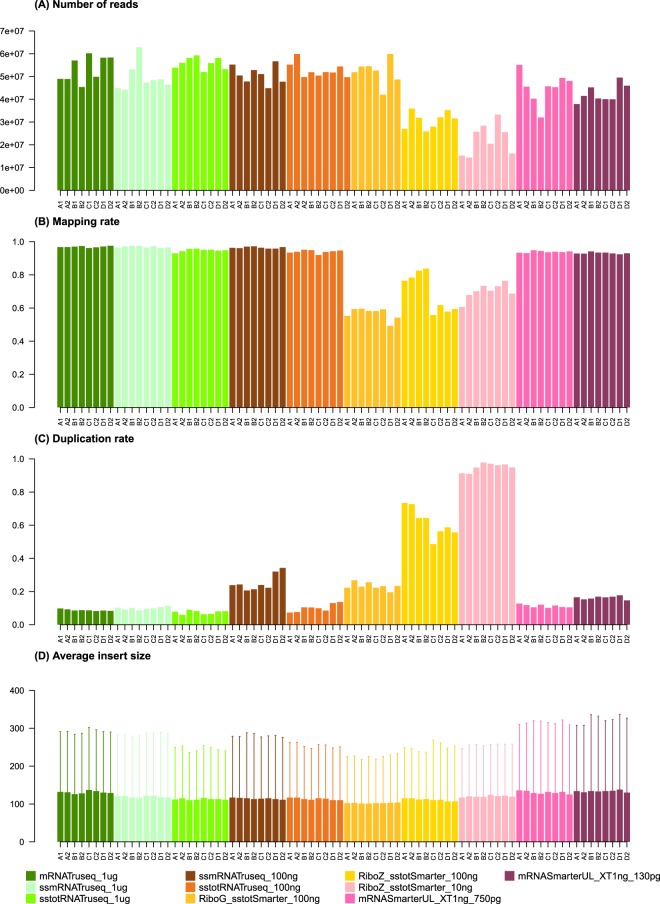
Read quality metrics for all samples and conditions. The metrics shown in the figure are the raw number of reads in the fastq files, the mapping rate after sequence trimming, the duplication rate and the average insert size.

Figure 1A shows the total number of reads sequenced for each sample. All the 1 *μ*g samples (mRNA TruSeq, stranded mRNA TruSeq and stranded total RNA TruSeq categories) display close values (average values per class of 53, 49 and 55 M reads respectively). The 100 ng samples give average values similar to the 1 *μ*g samples (average values of 50, 52 and 52 M mapped reads for stranded mRNA TruSeq, stranded total RNA TruSeq and RiboGone stranded total RNA SMARTer categories), with the exception of the RiboZero stranded total RNA SMARTer samples, which display a much lower output at an average of 31 M reads. The 10 ng input category (RiboZero stranded total RNA SMARTer) shows the lowest value with an average of 22 M reads. The two ultra-low samples, mRNA SMARTer UL 750 pg and 130 pg yielded a relatively high number of reads with an average of 45 and 42 M reads.

**Figure 2:**
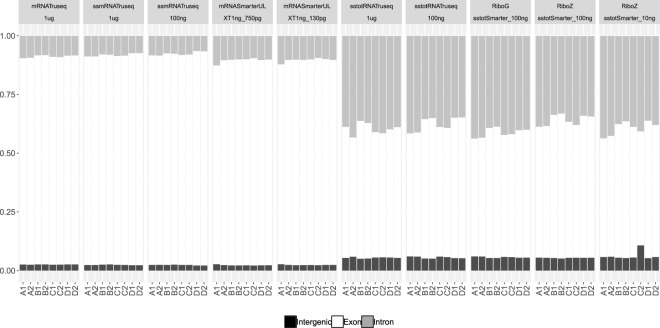
Normalized alignment rates (vertical axis) to intergenic, exon and intron regions for all the samples and conditions (horizontal axis). The mRNA samples are on located on the left side of the figure and the total RNA samples are on the right side.

The quality metrics mapping rate, duplication rate and average insert size displayed in Fig. 1 were all calculated for a uniform sampling level of 2 × 10 M. Figure 1B shows the mapping rate for the different categories. Not surprisingly, the three 1 *μ*g categories have very high mapping rates with an average percentage of 95–96% of all reads mapped. For the 100 ng samples, two categories show a high read mapping rate (stranded mRNA and total RNA TruSeq), while the other two categories show lower mapping rates (56% and 69% for the RiboGone and the RiboZero stranded total RNA SMARTer respectively). The 10 ng category shows an average mapping rate of 69%, while the two SMARTer ultra-low samples (750 pg and 130 pg) display much higher mapping rates at around 93%. The kits RiboGone SMARTer for 100 ng input total RNA, RiboZero SMARTer for 100 ng input total RNA and RiboZero SMARTer 10 ng input total RNA clearly show lower mapping rates than for the other conditions. Although the mapping rates for these three conditions are comparable but not exactly the same, the number of PCR cycles are the same for the three kits, suggesting the absence of a correlation. We checked the unmapped reads with fastQC^22^, but did not find any particular pattern that could easily explain these low mapping rates. This is most likely due to less efficient sample preparation for these kits. Mapping rates on ribosomal RNA sequences are available in Supplementary Table S1. Read quality metrics for transcriptome mappings are available in Supplementary Dataset S1. All the quality metrics values used in Figs 1 and 2 are available in Supplementary Dataset S4.

Duplication rate is shown in Fig. 1C. Unsurprisingly, the rates are very low for all the 1 *μ*g samples (around 8% on average for all three categories). As expected the duplication rates for the 100 ng samples are higher, however large variations in average values can be seen depending on the kit used. The lowest average rate is 10% for the category stranded total RNA TruSeq, followed by stranded mRNA TruSeq and RiboGone stranded total RNA SMARTer at 25% and 23% respectively. The last 100 ng group displays a very high rate at 61% (RiboZero stranded total RNA SMARTer). The 10 ng samples have an extremely high rate of duplication with an average of 94%. The two ultra-low sample groups have relatively low duplication rates with average values of 11% and 16% (750 pg and 130 pg respectively). However, caution should be taken in interpreting these values since the ultra-low kit has two consecutive steps for PCR amplification, and the values observed here represent duplication rates for the second stage only (see methods). In the previous paragraph, we noticed that the mapping rates are very low for the three Ribogone and Ribozero SMARTer kits, but their respective duplication rates are quite different, which does not suggest a relationship between these two metrics.

For the average insert size (Fig. 1D), we observed very little variation between the different categories, with an overall average insert size of around 110 nt.

Globally, the results show that the 1 *μ*g groups display the best results for quality metrics in terms of the high number of reads produced, as well as very high mapping rates and low duplication rates. No real distinction can be made at this level between the stranded versus unstranded or mRNA versus total RNA conditions. This group can be considered as the reference group in terms of performance and acceptable metrics levels. For the 100 ng input groups, the results are more contrasted. The two TruSeq groups (stranded mRNA and stranded total RNA) performed well, with a total number of reads and mapping rate comparable to that of the 1 *μ*g groups. Their duplication rates were higher than for the 1 *μ*g groups, although still in the acceptable ranges. The RiboGone stranded total RNA SMARTer group had a significantly lower mapping rate, while the last group, the RiboZero stranded total RNA SMARTer showed the lowest number of reads, a relatively low mapping rate and a very high duplication rate. Quality metrics for the 10 ng samples showed very poor results in all samples compared to the reference 1 *μ*g group. The number of reads is extremely low, the mapping rate is relatively low and the duplication rate very high. On the other hand, the metrics for the two ultra-low input amounts are reasonably good compared to the reference samples, with a relatively high number of reads and a very high mapping rate and low duplication rate, although this last metric should be interpreted cautiously for this group, for the reasons mentioned above. Read quality metrics analysis showed that rRNA depletion methods (RiboGone and especially RiboZero) have a negative effect on RNA-seq quality. The rRNA depletion method was efficient in removing rRNA and tRNA, but was less efficient than a polyA selection method. Notably, it involves one more purification step than for polyA selection and the method loses more RNA of interest. However, rRNA depletion methods are able to give access to other types of RNA molecules (such as long non-coding RNAs or nascent RNAs) because of the use of random primers for the reverse transcription step, and these methods could also be more efficient than polyA selection in cases of low quality RNA (for example, a RIN score <6).

Figure 2 shows the normalized rates of mapping to intergenic, exonic and intronic regions of the genome. As might be expected, there is a clear distinction between the mRNA and total RNA samples. The mRNA samples all map predominantly to exonic regions, then to intronic and finally to intergenic regions. On the other hand, for the total RNA samples an almost equal proportion of reads map to the exonic and intronic regions, and considerably less reads map to the intergenic regions (although this smaller proportion is still higher than for the mRNA samples). All the mRNA samples have similar proportions, with a slight decrease in the exonic regions for the ultra-low (SMARTer UL) groups. In the total RNA samples, the rates are very similar for the different kits and input amounts.

### Gene detection and counting

**Figure 3:**
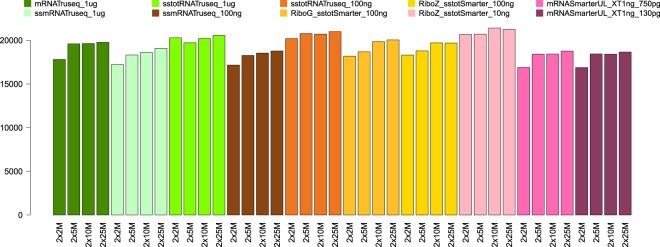
Number of genes detected (vertical axis) for all sampling levels and all conditions (horizontal axis).

**Figure 4:**
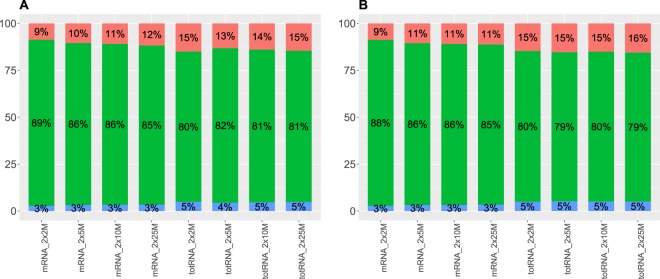
Percentage of pseudogenes (blue), protein-coding (green) and non-coding RNAs (red) for 1 *μ*g TruSeq samples (**A**) and 100 ng TruSeq samples (**B**). The percentages are indicated within the bars. The vertical axis indicates the percentage and the horizontal axis indicates the sample type and sampling level. In each figure, the mRNA samples are on the left side and total RNA samples are on the right.

**Figure 5:**
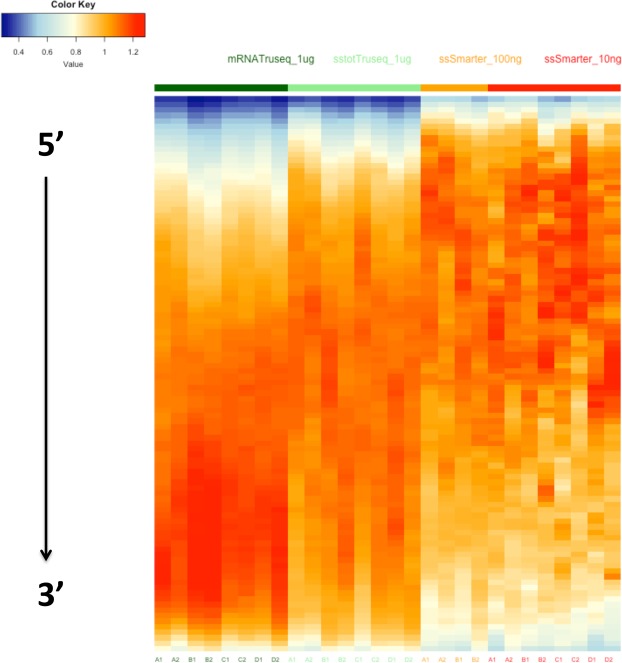
Heatmap of the coverage percentage for 1,000 genes having a medium expression level for the standard and low input amounts categories. The coverage values are standardized to take into account the different gene lengths.

Figure 3 shows the number of genes detected for each sequencing protocol and sequencing depth (2 × 2, 2 × 5, 2 × 10 and 2 × 25 M reads). We used a low threshold whereby a gene was considered expressed if it had a CPM (counts per million) value greater than or equal to one in two replicates (see methods). Overall, the different groups showed similar values for the number of genes detected. As also shown in Fig. 3, the number of detected genes increases with the sequencing depth for all the categories, and the 2 × 2 M sequencing depth category has the lowest number of detected genes in all the groups. Only the ultra-low input samples show a slight decrease in the number of genes detected. Note that if we take the top 5,000 expressed genes in each condition, the number of genes common to all conditions is equal to 1,854. If we take the standard input amount and the low input amount categories shown in Fig. 4A,B, we can see that the proportion of non-coding RNAs and pseudogenes is higher for the total RNA groups. This is easily explained by the fact that non-coding transcripts are much better covered by total RNA protocols. The proportions of protein-coding, non-coding and pseudogenes are similar for the low and ultra-low input amount samples (10 ng stranded total RNA SMARTer and mRNA SMARTer UL 750 and 130 pg, see Supplementary Fig. S1). Figure 5 shows the normalized percentage of mapped reads from the 5′ end to the 3′ end of the genes. For the mRNA Truseq 1 *μ*g, we clearly see a coverage bias towards the 3′ end of the genes. This is a known fact and is a consequence of the polyA selection for these samples. The profiles are much more balanced for the stranded total RNA TruSeq, a consequence of the random priming process. For the low input quantities (100 ng and 10 ng), we can see that the coverage is much more random due to the dilution and a random priming of the reads along the gene. Gene counts for 0.1, 1 and 10 CPM (counts per million) thresholds are displayed in Supplementary Table S2 for the reference sample unstranded mRNA TruSeq 1 *μ*g. A ROC curve showing the true detection rate as a function of the false detection rate for all input quantities and preparation kits at the 2 × 25 M sampling level is given in Supplementary Fig. S2. The Supplementary Fig. S3 shows scatter plots and correlation values between detected genes for all conditions at the 2 × 25 M sampling level and detected genes for the reference condition unstranded mRNA TruSeq 1 *μ*g at the 2 × 25 M sampling level.

### Differential expression

**Figure 6:**
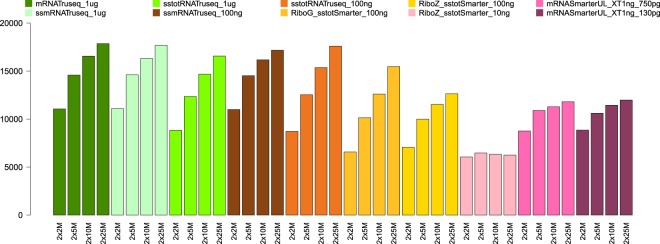
Number of differentially expressed genes (DEG, vertical axis) detected between samples A and B for all sampling levels and conditions (horizontal axis).

In this section, we evaluate the impact of the different kits and input amounts on a typical differential gene expression analysis. For each condition, we compute the list of differentially expressed genes (DEG) between samples A and B (with two technical replicates for each sample) with EdgeR^23^, at a given sampling depth (2 × 2 M reads, 2 × 5 M reads, 2 × 10 M reads and 2 × 25 M reads) and for a given corrected p-value threshold. We obtained, as a result, a list of DEG that can be compared between the different conditions. Figure 6 shows the number of differentially expressed genes (DEG) in sample A (mixture of normal human tissues) and B (human brain tissues) replicates. For the standard input unstranded mRNA samples, a minimum of 11,059 DEG can be seen for the 2 × 2 M sampling level and a maximum of 17,855 DEG for the 2 × 15 M sampling level. The number of DEG is similar for the standard stranded mRNA, while a slight decrease can be observed for the 1 *μ*g total RNA samples: this is visible at all sampling levels. For the low input (100 ng) samples, the DEG profiles observed for the stranded mRNA and total RNA TruSeq are very similar to those of their standard counterparts. In contrast, there is a clear decrease in the number of DEG detected for the low input amount (100 ng) RiboGone and RiboZero stranded SMARTer samples, at all sampling levels. Out of all the groups, the low input (10 ng) RiboZero stranded SMARTer samples clearly show the lowest number of DEG for all sampling levels. The number of DEG for the ultra-low samples is clearly higher at all sampling levels compared to the low input (10 ng) RiboZero stranded total RNA SMARTer samples. The number of DEG in the ultra-low samples is comparable to that of the low input (100 ng) RiboZero stranded total RNA SMARTer, with even higher levels observed for the sampling levels 2 × 2 M and 2 × 5 M. Overall, despite their very low input amounts, the ultra-low samples performed reasonably well for the detection of DEG. Note that if we take the standard rate of 5% FDR (false discovery rate) level, we obtain a total of 2,463 DEG common to all the conditions. We calculated Gene Ontology biological processes enrichment for this list of genes using the enrichR tool^24^. We found a total of 285 biological processes categories enriched with a p-value < 0.05. The complete list of categories with p-values, adjusted p-values, proportions, scores and list of genes associated to each category is available in Supplementary Dataset S3. Many of the top enriched categories are related to the central nervous system, such as “central nervous system development”, “axonogenesis”, “glutamate receptor signaling pathway”, “neuron projection morphogenesis” or “neurotransmitter transport”. This is expected, since the B sample is composed of various brain tissue extracts while the A sample is a mixture of various tissues (including some brain tissues). There are nonetheless other types of biological processes categories enriched, such as “cytoskeleton organization” or “elastic fiber assembly”.

Table 2 shows an overlap in the number of DEG between the unstranded mRNA TruSeq 1 *μ*g samples used as the reference set and a selection of kits/input amounts. The overlap with the reference set is highest for the 1 *μ*g input amounts (91% and 79%), but is still relatively high for the 100 ng categories (89% and 84%), and, as expected, is lower for the ultra-low input amount (54% and 55%) but still above 50%. This is not the case for the 10 ng RiboZero stranded total RNA SMARTer which falls below this level to 26%, thus much lower than the ultra-low samples. The complete list of DEG with ensembl gene IDs, log fold change, log CPM, p-values and FDR for mRNATruseq_1 ug at 2 × 25 M sampling depth is available in Supplementary Dataset S2.

**Table 2:** Overlap in the number of differentially expressed genes (DEG) between a reference set (mRNATruseq_1 ug) and all the other conditions for the sampling level of 2 × 25 M.

Set 1	Set 2	NDEG Set 1	NDEG Set 2	Inter. S1 S2	%
mRNATruseq_1 ug	ssmRNATruseq_1 ug	16983	16942	15505	91%
mRNATruseq_1 ug	ssTotRNATruseq_1 ug	16983	15181	13457	79%
mRNATruseq_1 ug	ssmRNATruseq_100 ng	16983	16305	15070	89%
mRNATruseq_1 ug	ssTotRNATruseq_100 ng	16983	16367	14248	84%
mRNATruseq_1 ug	RiboG_ssTotSmarter_100 ng	16983	13702	12285	72%
mRNATruseq_1 ug	RiboZ_ssTotSmarter_100 ng	16983	10677	10013	59%
mRNATruseq_1 ug	RiboZ_ssTotSmarter_10 ng	16983	4562	4419	26%
mRNATruseq_1 ug	mRNASmarterUL_XT1 ng_750 pg	16983	9671	9233	54%
mRNATruseq_1 ug	mRNASmarterUL_XT1 ng_130 pg	16983	9775	9330	55%

The percentage represents the fraction of DEG relative to the reference set.

### Gene expression accuracy

**Figure 7:**
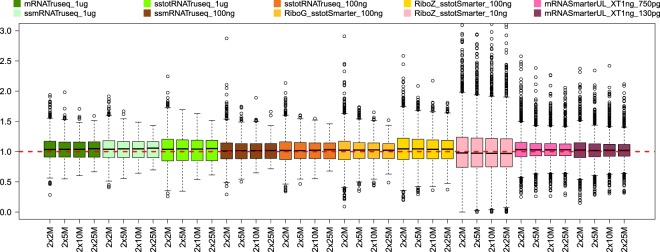
Ratios (vertical axis) between real and predicted gene expression values for samples C for all sampling levels and conditions (horizontal axis). The red dotted line indicates a ratio value of 1, i.e. a perfect match between real and predicted value. Some of the outlier values are not shown in this figure.

**Figure 8:**
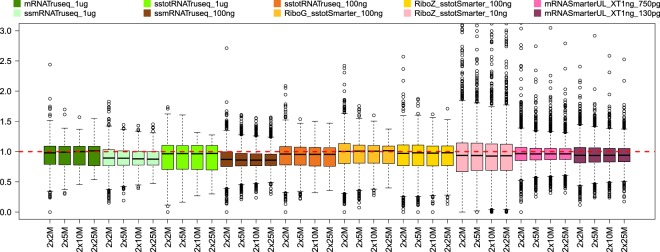
Ratios (vertical axis) between real and predicted gene expression values for samples D for all sampling levels and conditions (horizontal axis). The red dotted line indicates a ratio value of 1, i.e. a perfect match between real and predicted value. Some of the outlier values are not shown in this figure.

The samples labelled C and D are a mixture of the Human Universal Reference Total RNA (A) and Human Brain Reference RNA (B), with ratios of 75% A + 25% B and 25% A + 75% B respectively. Since we have the gene expression levels for samples A and B, we can predict the theoretical gene expression values for the mixture samples C and D and compare these to the values obtained by analyzing the samples. Thus we can evaluate the accuracy of the gene expression levels in samples C and D for all conditions tested. The boxplots in Figs 7 and 8 show the gene expression level ratios between the real and predicted values for samples C and D respectively. The red dotted line in the figures indicates the perfect agreement (ratio value of 1) between the real and the predicted values. For the sample C ratios (Fig. 7), in most cases the median value for each group is aligned or very close to the red dotted line, indicating very good agreement between the predicted and real gene expression values. For the sample D ratios, the agreement is globally good, but as can be seen from the figure there are larger deviations from the red dotted line, for instance for the standard input TruSeq stranded total RNA, the low input (100 ng) TruSeq stranded mRNA group, the low input (10 ng) RiboZero stranded total RNA SMARTer group and the ultra-low input (130 pg) mRNA SMARTer UL. We also clustered samples A, B, C and D according to the normalized counts (Supplementary Fig. S4) and in most cases, the samples clustered logically, showing the C samples to be more closely related to the A samples, and the D samples to be more closely related to the B samples. Furthermore, mRNA samples and Total RNA samples also cluster together, as well as A, B, C and D samples. Interestingly, we can see on the figure that several RiboZero stranded total RNA Smarter samples with 10 ng input amount do not cluster well with the other samples and form outliers.

## Discussion

Following global cost reductions for sequencing, RNA-seq is now an affordable and popular technique in various areas of biological research, with potential applications in clinical medicine^1^. For instance, RNA-seq can be applied in the clinical context for the detection of aberrant transcription in human diseases, notably in the cases of aberrant expression profiles, gene fusion expression, alternative transcripts and allele-specific expression^1^. However, robust gene expression analysis and other potential clinical applications of RNA-seq are dependent on the quality, reliability and reproducibility of the extraction and sequencing procedures. In this study, we performed a comprehensive assessment of several state-of-the-art and popular commercial kits for RNA-seq library preparation kits using standard, low and ultra-low input amounts. We assessed the performance of the kits using various sequencing and read alignment metrics, gene expression detection levels and gene differential expression levels at various sampling depths. The results are summarized in Table 3, which indicates the global levels of quality for the different metrics analyzed in this study. We have also included an indicator of the automation capability of each kit, i.e. to indicate whether or not the kit can be used with liquid handling robotic systems. This capacity can be important for the reproducibility and robustness of the results, in the case of large research projects or in sequencing centers that process large numbers of samples.

**Table 3:** Assessment summary of the different RNA preparation kits and conditions analyzed in this study.

Kit/Input	NR	MR	DR	GD	DEG	AC
**Illumina technology**						
mRNATruseq_1 ug	*****	*****	*****	*****	*****	Yes
ssmRNATruseq_1 ug	*****	*****	*****	*****	*****	Yes
ssTotRNATruseq_1 ug	*****	*****	*****	*****	*****	Yes
ssmRNATruseq_100 ng	*****	*****	***	****	*****	Yes
ssTotRNATruseq_100 ng	*****	*****	*****	*****	*****	Yes
**SMARTer technology**						
RiboG_ssTotSmarter_100 ng	****	*	***	****	***	Yes
RiboZ_ssTotSmarter_100 ng	**	**	**	***	**	No
RiboZ_ssTotSmarter_10 ng	*	**	*	***	*	No
**SMARTer Ultra-Low technology**						
mRNASmarterUL_XT1 ng_750 pg	***	****	NA	***	***	Yes
mRNASmarterUL_XT1 ng_130 pg	***	****	NA	***	***	Yes

The table presented here is based on the 2 × 10 M sampling level. The number of stars indicates the global level of quality of the metrics used, with a maximum of five stars for best quality. NR, MR and DR: sequencing and alignment quality metrics (Number of reads, mapping rate and duplication rate). GD: gene detection metrics. DEG: differentially expressed gene metrics. AC: automation capability.

## Conclusion

The standard input amount (1 *μ*g) TruSeq unstranded mRNA kit can be considered as the reference material or the “gold standard” in this study, as this kit has been very widely tested and used for RNA-seq studies, and the amount of input material is usually considered more than enough for most RNA-seq applications. The other kits used for the standard input amount (stranded TruSeq mRNA and total RNA) perform equally well in terms of the sequencing and alignment metrics, the number of detected genes and the number of genes differentially expressed. For the low input amounts (100 ng), the TruSeq kits (stranded mRNA and total RNA) achieve similar performances, except for a slightly elevated duplication rate for the TruSeq stranded mRNA. The other kits tested for low input, i.e. the RiboZero or RiboGone stranded total RNA associated with the SMARTer kit, clearly show decreased performance on multiple levels compared to the TruSeq kits. For the low and ultra-low input amounts, the stranded RiboZero SMARTer kit with the 10 ng input amount underperforms on most of the indicators. Note that at this 10 ng input amount, we tested only one kit with the rRNA depletion method RiboZero. It would be informative to test other rRNA depletion methods, such as the RiboGone, as well as other kits compatible with this input amount. In contrast, the ultra-low unstranded mRNA SMARTer samples, with single cell-like input quantities, achieve very good levels for most of the performance indicators, and particularly for the sequencing and alignment quality metrics. In summary, our study shows that Illumina TruSeq technology performs very well for standard and low input amounts of both mRNA and total RNA on all metrics measured, and furthermore it has good automation capabilities (see Table 3 for a summary of all indicators). The SMARTer technology tested here for low input quantities, clearly displays much lower overall performances, and when associated with the RiboZero step it is not adapted to automation. Thus, we would not advise choosing this technology for the conditions tested here. On the other hand however, for ultra-low input amounts, the SMARTer ultra-low technology (combination of SMARTer UL and Nextera) tested here shows good performance indicators on multiple levels despite the ultra-low input, and it is also adapted to automation. Therefore we would recommend the use of this technology for ultra-low sample input amounts. It is worth noting that although we did not test this condition in the present study, the SMARTer ultra-low technology can be used with input amounts up to 10 ng, thus with a range of use from 10 ng down to single-cell input conditions.

## Methods

### Samples

Two commercial RNA samples were used to evaluate the different RNA-seq applications: 1) Human Universal Reference Total RNA (Takara Bio/Clontech, labeled A), a mixture of 23 normal human tissues (including brain), and 2) First Choice Human Brain reference RNA (Ambion), a pool of normal human brain tissues (labeled B). The other samples were prepared as follows: C = 75% A + 25% B; D = 25% A + 75% B. All the samples were prepared in duplicate for all the RNA applications. In addition, all samples were prepared from the Human Universal Reference Total RNA and the First Choice Human Brain reference RNA samples and were qualified on the BioAnalyzer before being pooled and labelled A, B, C and D. Supplementary Table S3 shows the RIN scores for the original samples as well as the samples A, B, C and D. All libraries were loaded at 8 pM. Because of the nucleotide imbalance at the beginning of first read of the Smarter libraries (10 ng and 100 ng input), the samples were carefully pooled before loading on the sequencers with balanced libraries from others experiments. The BioAnalyzer traces for the libraries from sample A are provided in Supplementary Fig. S5. The main specific steps for library preparation are displayed in Supplementary Fig. S6. All the samples were sequenced in duplicates, with two technical replicates created from the sample preparation. The catalog numbers for the kits used in this study are provided in Supplementary Table S4.

### RNA-seq protocols

#### Illumina TruSeq technology

Three kits from Illumina were tested with different total RNA input: TruSeq mRNA with an input amount of 1 *μ*g, TruSeq stranded mRNA with inputs of 1 *μ*g and 100 ng and TruSeq stranded total RNA Gold with 1 *μ*g and 100 ng. The TruSeq mRNA and stranded mRNA kits use polyA selection to trap the mRNA before proceeding to the cDNA synthesis. In the case of the TruSeq stranded total RNA Gold kit, the RiboZero Gold technology targets the rRNA with baits, and the cDNA synthesis takes place after purification. To ensure that the information on the strand is kept in the experimental procedure, the stranded kits use both actynomycin-D (during the first strand cDNA synthesis) and dUTP (during the second strand synthesis). The libraries are end-repaired and adenylated before being ligated with Y-shape single indexed adaptors, and then are amplified by PCR. We followed the Illumina recommendations for all the kits, except for the last purification step which we performed with Ampure XP beads at 0.8X to remove all the adapter dimers.

#### Stranded SMARTer technology

Starting with mRNA or ribosomal depleted RNA material, the stranded SMARTer technology (Takara Bio/Clontech) is based on tagged random hexamer primers and a SMARTScribe Reverse Transcriptase (RT) with terminal transferase activity. When it finishes the first strand cDNA synthesis, the RT adds a few non-templated nucleotides to the 3′ end of the cDNA (GGG). The SMART adapter is complementary to these nucleotides and adds the 5′ tag. The first strand cDNA is used as a matrix to perform the PCR amplification using indexed primers. In order to remove the rRNA from the input, we tested the RiboZero Gold kit (Illumina) and the recently released RiboGone Mammalian kit (Takara Bio/Clontech). The latter combines hybridization of rRNA and mtRNA baits with RNAse H digestion. A combination of RiboZero plus SMARTer stranded was used with 10 ng and 100 ng of total RNA input. Purification was performed on purifying columns (Macherey Nagel) before using the SMARTer stranded kit. We also tested a combination of RiboGone plus SMARTer stranded with an input of only 100 ng. We followed the Takara Bio/Clontech recommendations and the number of PCR cycles that we performed is indicated in Table 1.

#### SMARTer Ultra Low technology

The SMARTer Ultra Low technology (Takara Bio/Clontech) is also based on the RT properties but uses polydT primers to synthesize the first strand of the cDNA. The full-length cDNA is then amplified by LD-PCR before being quantified with the High Sensitivity Qbit kit and qualified with the High Sensitivity chip in a BioAnalyzer machine. We normalized in 2 different quantities: 130 pg and 750 pg were used as input for the Nextera XT kit (Illumina). The Nextera technology uses a modified tranposase to fragment and ligate adapters in a unique step called “tagmentation”. The libraries are then amplified by PCR using indexed primers. Full details of the LD-PCR and PCR are given in Table 1. The last purification step is performed with a ratio of 0.6X of XP Ampure Beads.

#### Sequencing

The qualification and quantification estimations for each library were done after the last purification using DNA1000 chip (input ≥ 100 ng) or High Sensitivity chip (input < 100 ng) for BioAnalyzer (Agilent). After normalization the libraries were sequenced in 4-plex on HiSeq 2000 or HiSeq 2500 machines (Illumina) in paired-end 2 × 100 bp.

### Quality check, alignment and quantification

After sequencing, read quality was checked with fastQC^22^ (version 0.11.3) and trimmed with a quality threshold of 30 using trimmomatic^25^ (version 0.32). The reads were then aligned against the Ensembl human reference genome GRCh37 with Tophat2^26^ (version 2.0.13). We used the Picard-Tools^27^ (version 1.138) module CollectRNASeqMetrics to calculate the mapping rate, the duplication rate and the average insert size. Finally, we used htseq-count^28^ (version 0.6.1) to assign and count the reads for all the Ensembl GRCh37 gene models.

#### Gene expression detection

Expression detection sensitivity was based on the analysis of the two technical A replicates for each condition. All genes for which both CPM (counts per million) of normalized expression were greater than 1 were considered as expressed. In other words, we declared as detected all genes for which:

$${1}_{\{{x}_{cpm}^{A1}\ge 1\}}+{1}_{\{{x}_{cpm}^{A2}\ge 1\}}=2$$

#### Differential gene expression

All statistical analyses were performed with the R software^29^ (version 3.4.3). Differential expression between the two HsRef replicates (A) and the two Brain replicates (B) was studied using the R package EdgeR which implements a TMM normalization and a negative binomial approach as described by Robinson *et al*.^23^. We removed from the analysis all genes for which all CPM values (for the four samples) were lower than 1. To take into account the multiple testing problem that arises when testing a large number of genes simultaneously, we applied the Benjamini-Hochberg procedure (BH) at a 5% threshold.

It is worth noting that we also considered the two other popular approaches for differential analyses, DESeq 2^30^ and voom+Limma^31^, that lead to similar results.

#### Gene expression accuracy

To evaluate the accuracy of each protocol, we considered only genes which were declared as differentially expressed for all protocols in the previous step. To take account of differences in gene length, we normalized the raw counts using the FPKM approach. Predicted values ($\hat{C}$ and $\hat{D}$) were calculated as the weighted sum of the averages of the two replicates for A and for B:

$$\hat{C}=\frac{3}{4}\bar{A}+\frac{1}{4}\bar{B}$$

and

$$\hat{D}=\frac{1}{4}\bar{A}+\frac{3}{4}\bar{B}$$

For each gene, we then calculated the difference between the mean (over the two replicates) of the observed values for the mixture C (resp. D) and the predicted values $\hat{C}$ (resp. $\hat{D}$). We calculated the Euclidean distance between the average normalized measurements for C (resp. D) and the predicted values $\hat{C}$ (resp. $\hat{D}$). Finally, we performed hierarchical clustering using all genes with Euclidean distance and complete linkage, to determine whether similar samples clustered together.

### Accession codes

All the raw data and the gene count files are available in the GEO repository under accession code GSE124198.

## Supplementary information

Supplementary Information

Dataset S1

Dataset S2

Dataset S3

Dataset S4
