# Supplementary material for: Systematic analysis of TruSeq, SMARTer and SMARTer Ultra-Low RNA-seq kits for standard, low and ultra-low quantity samples
Source: Sci Rep. 2019 May 17;9:7550. doi: 10.1038/s41598-019-43983-0 (PMC6525156; doi:10.1038/s41598-019-43983-0)
Supplement: Supplementary file 1 — Supplementary Information [file 41598_2019_43983_MOESM1_ESM.pdf]

Systematic analysis of TruSeq, SMARTer and SMARTer Ultra-Low RNA-seq kits for standard, low and ultra-low quantity samples

Marie-Ange Palomares, Cyril Dalmasso, Eric Bonnet, Céline Derbois,
Solène Brohard-Julien, Christophe Ambroise, Christophe Battail,
Jean-François Deleuze, and Robert Olaso

Supplementary Figures and Tables

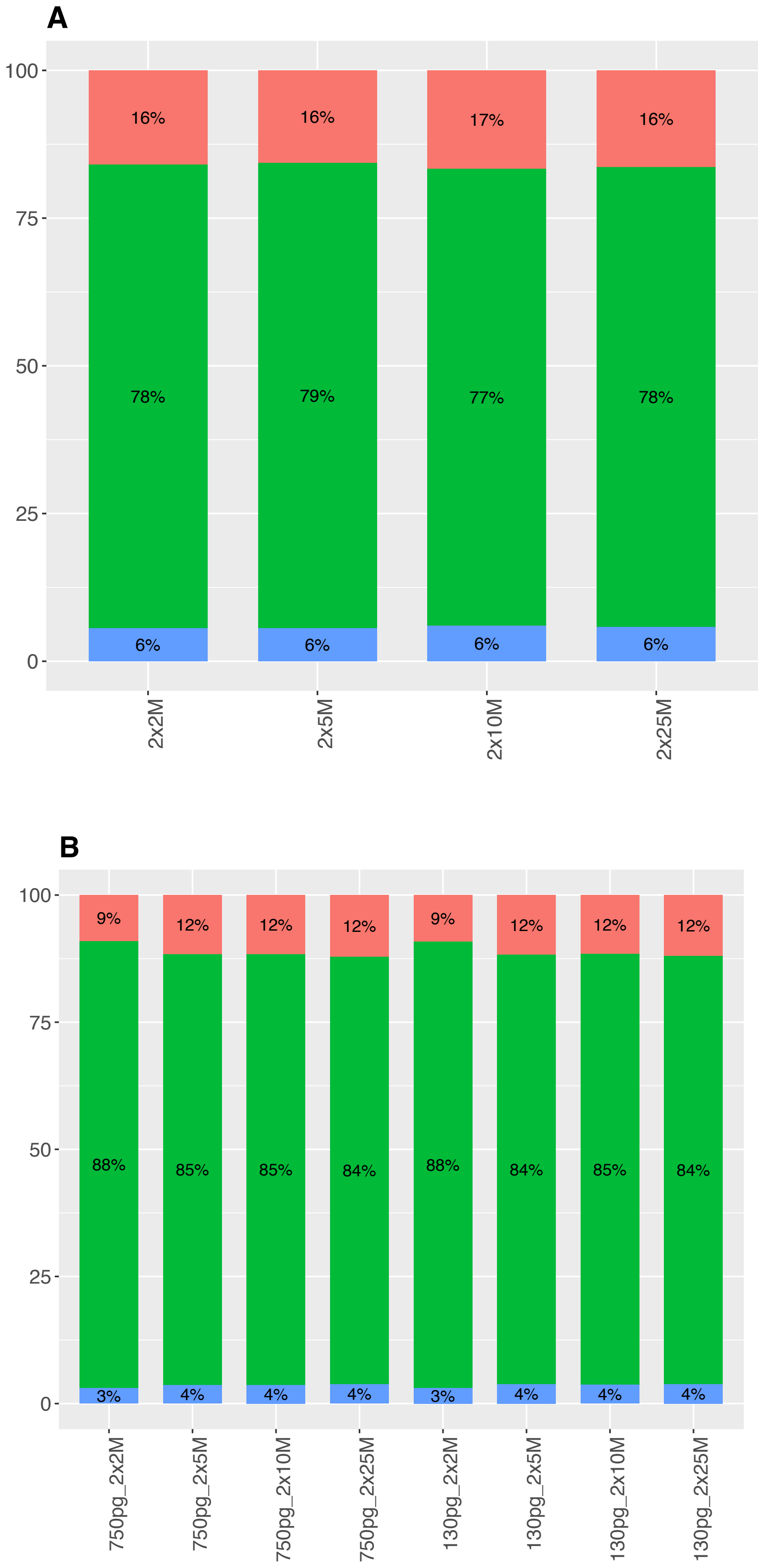

Supplementary Figure S1: Percentage of pseudogenes (blue), protein-coding (green) and non-coding RNAs (red) for stranded total RNA SMARTer with 10ng input quantity (A) and mRNA SMARTer UL XT with 750 pg and 130pg (B).

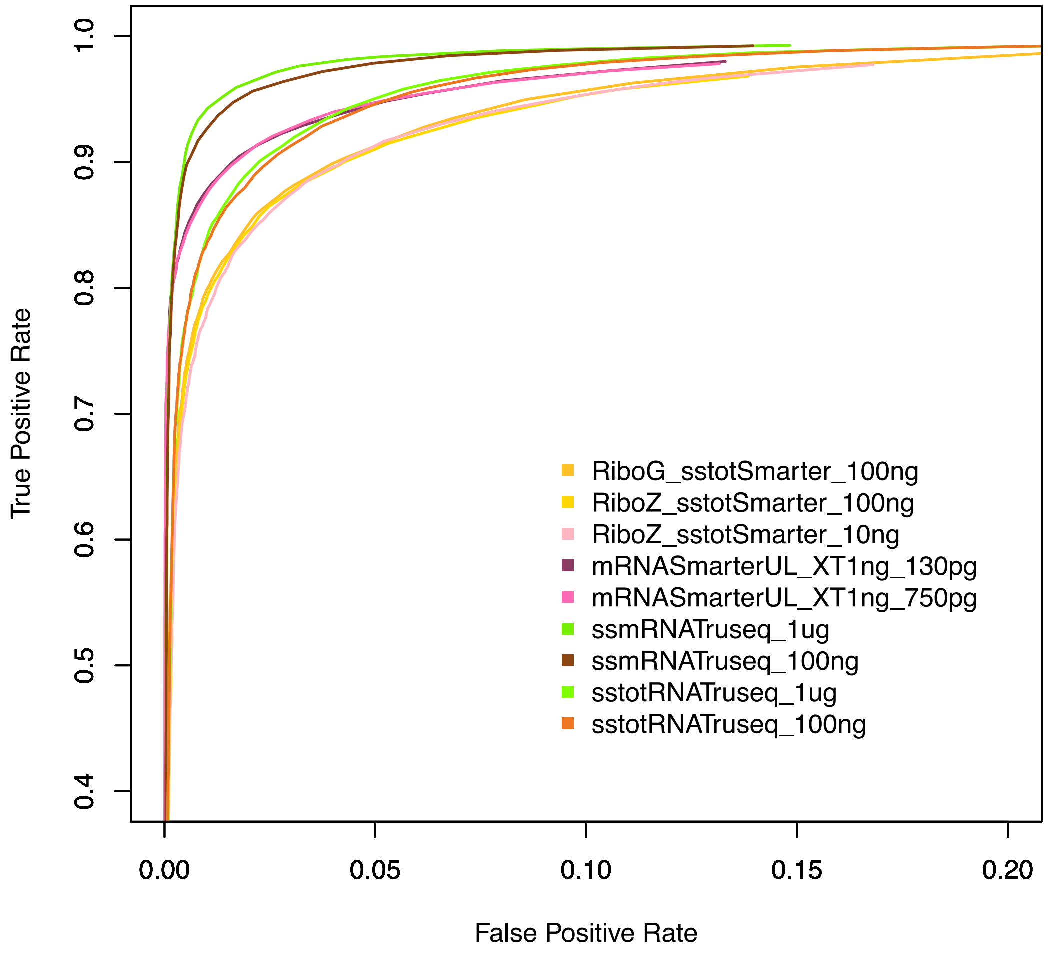

Supplementary Figure S2: ROC (Receiver Operating Characteristic) curves, plotting the false positive rate versus the true positive rate for detected genes. The ROC curves display the true detection rate as a function of the false detection rate for all input quantities and preparation kits at 2x25M sampling level. A grid of thresholds from 0.1cpm to 200 cpm was considered. The true set of detected genes was defined from the unstranded mRNA TruSeq 1 ug at 2x25M sampling level dataset with threshold 1 cpm.

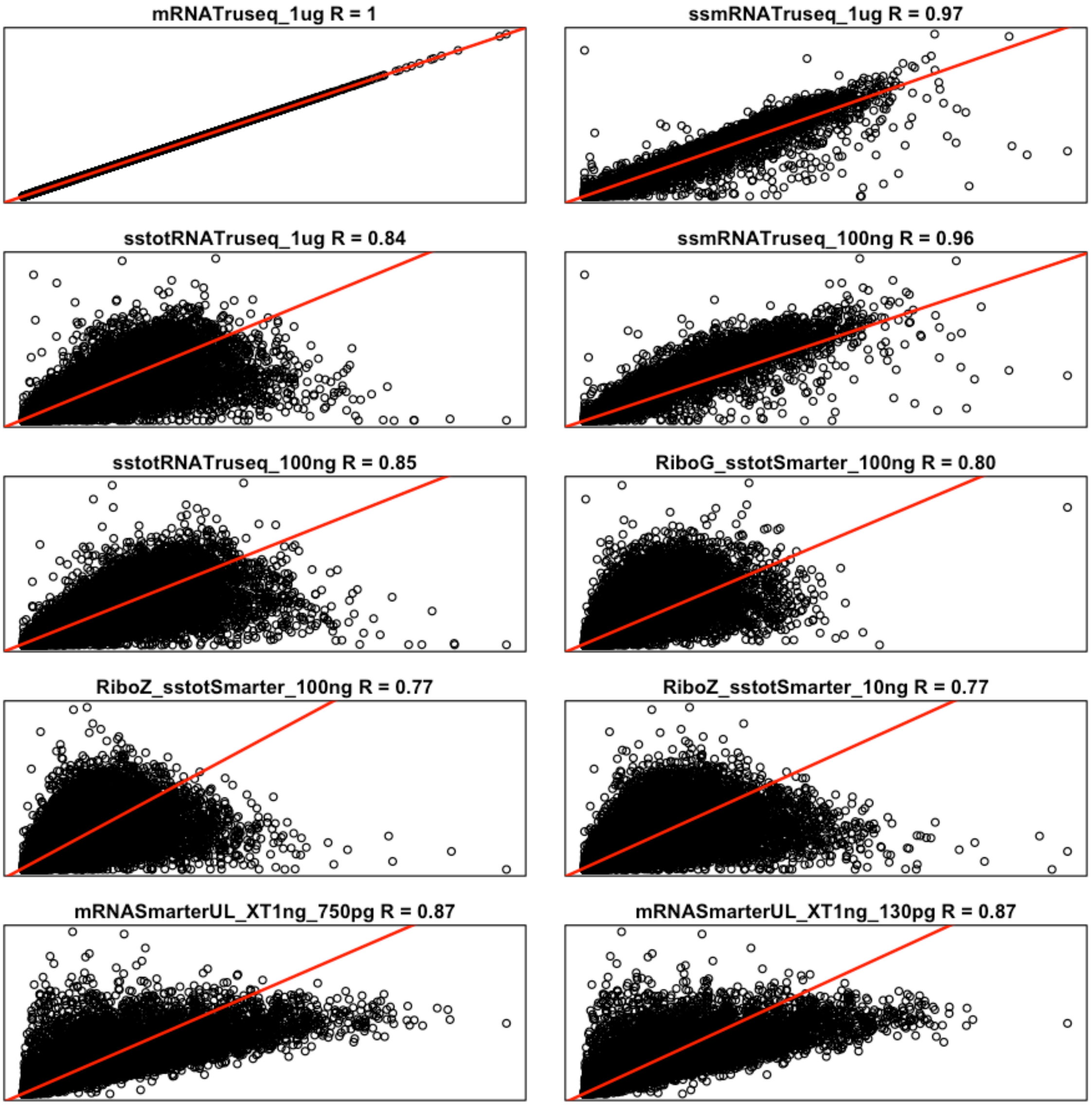

Supplementary Figure S3: scatter plots and correlation coefficient values (indicated in the title) between detected genes for all conditions at 2x25M sequencing depth and detected genes from the reference set mRNATruseq_1ug at 2x25M. The red line represents a linear regression between the two vectors. The y axis always represent the reference set.

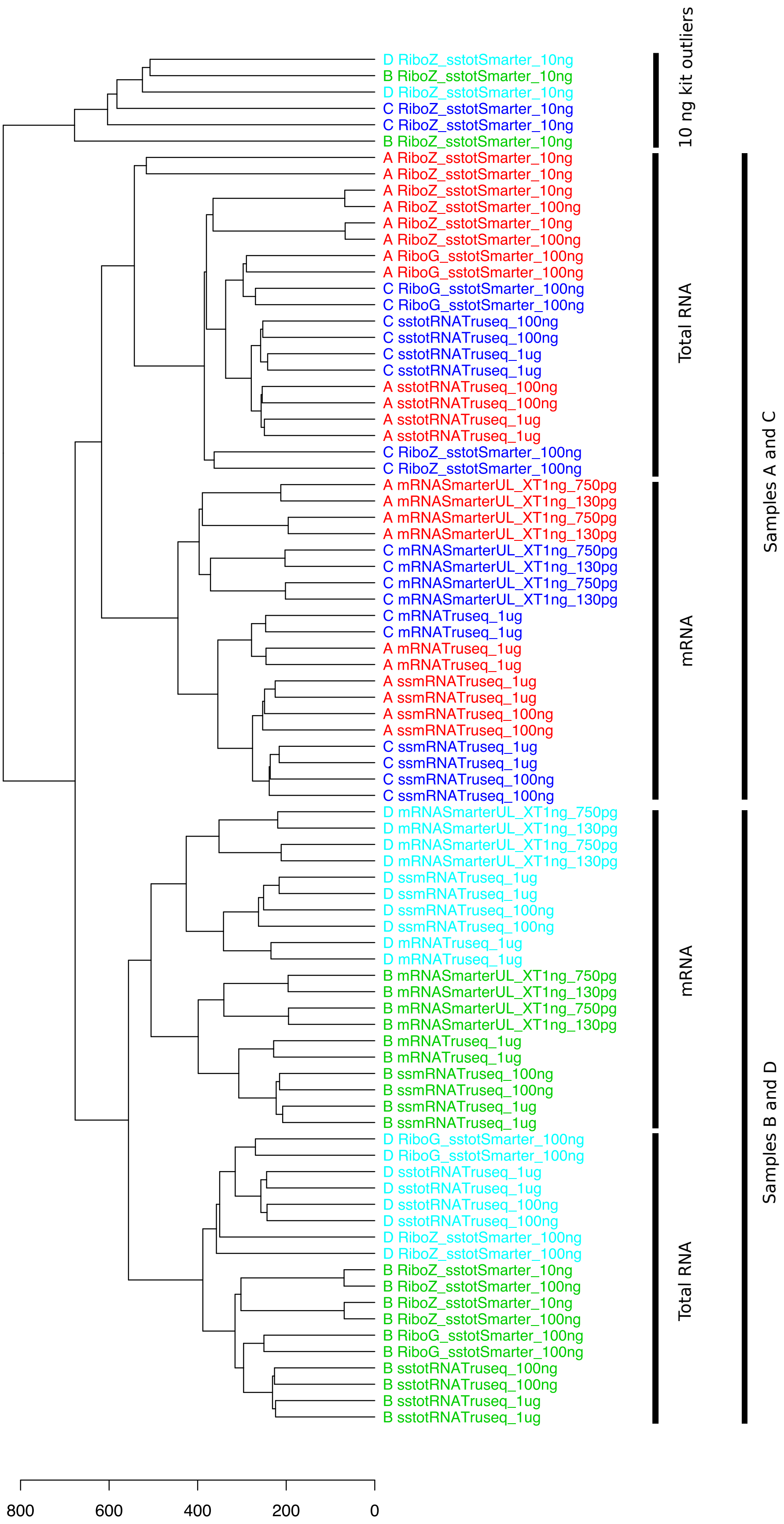

Supplementary Figure S4: hierarchical clustering of samples A, B, C and D from gene counting data for all the conditions.

Experiments	Samples	Technical replicate
mRNA Truseq 1ug (DNA1000)	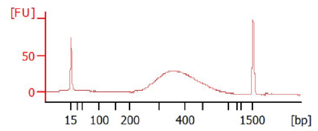	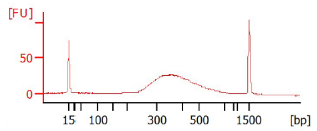
Ss mRNA Truseq 1ug (DNA1000)	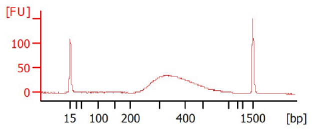	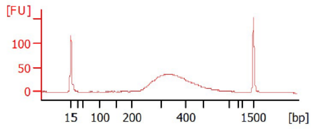
Ss totRNA Truseq 1ug (DNA1000)	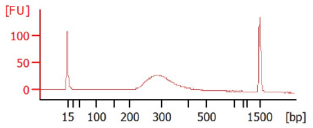	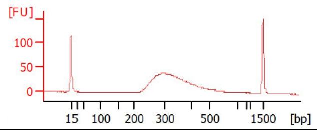
Ss mRNA Truseq 100ng (DNA1000)	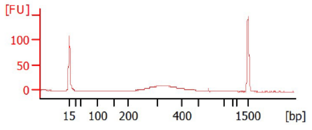	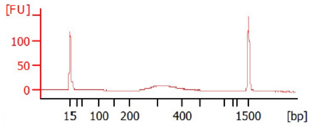
Ss totRNA Truseq 100ng (DNA1000)	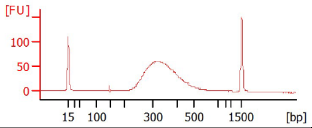	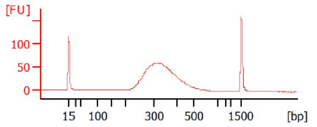
RG Ss totRNA Smarter 100ng (HS) Dilution 1/4	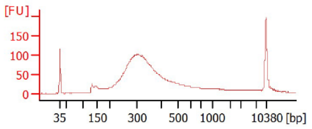	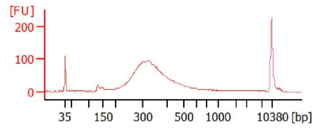
RZ Ss totRNA Smarter 100ng (HS)	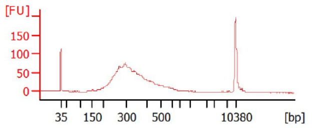	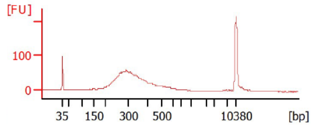
RZ Ss totRNA Smarter 10ng (HS)	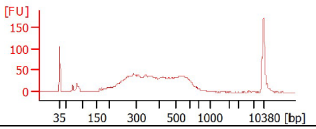	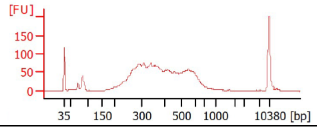
Smarter UV + Nextera XT 1ng/750pg (HS)	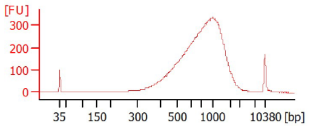	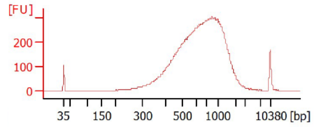
Smarter UV + Nextera XT 1ng/130pg (HS)	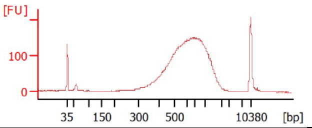	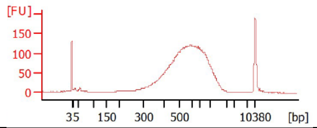

Supplementary Figure S5: BioAnalyzer traces (on DNA1000 and High Sensitivity chips) of the libraries from sample A.

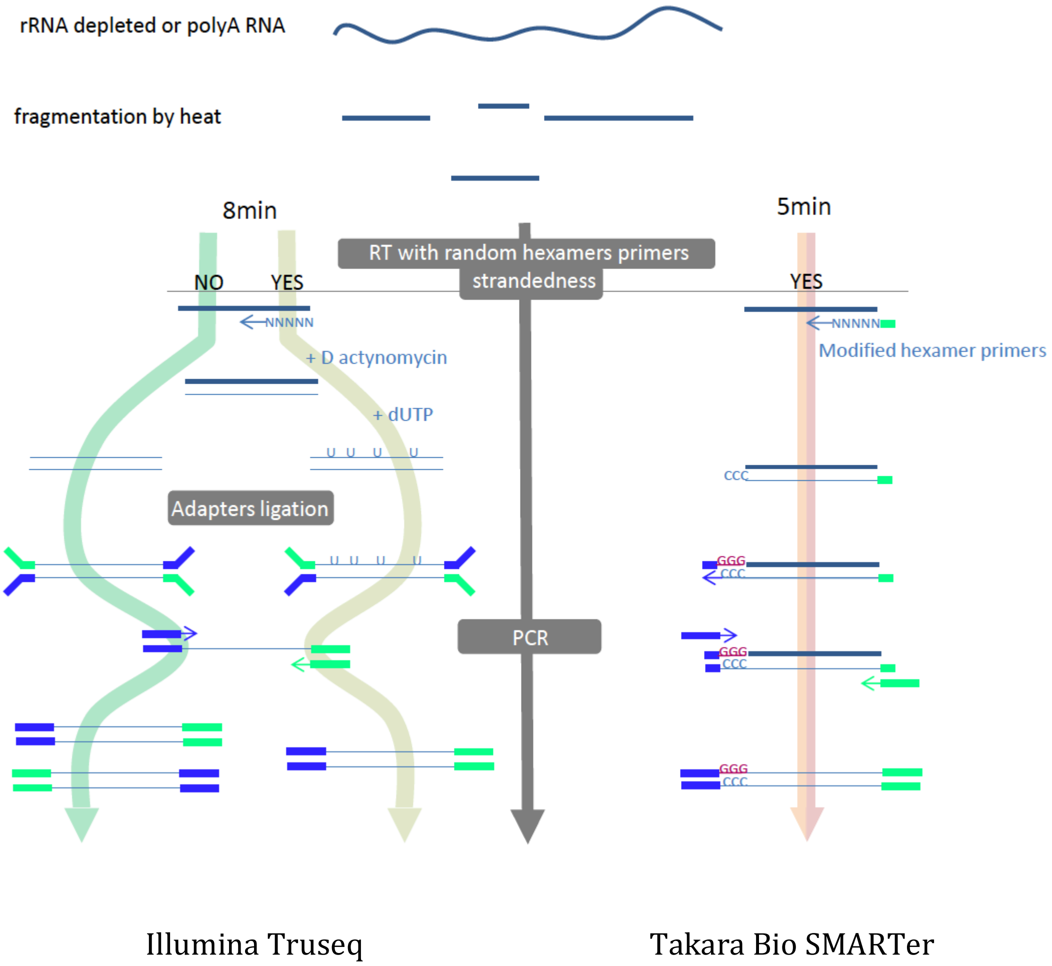

Supplementary Figure S6: scheme of the relevant specific steps for library preparation for the two main technologies used in this study, the Illumina Truseq and the Takara Bio SMARTer.

Condition	Sample	rRNA mapping %
RiboG_sstotSmarter_100ng	A1	8
RiboG_sstotSmarter_100ng	A2	8,43
RiboG_sstotSmarter_100ng	B1	8
RiboG_sstotSmarter_100ng	B2	7,62
RiboG_sstotSmarter_100ng	C1	8,74
RiboG_sstotSmarter_100ng	C2	8,36
RiboG_sstotSmarter_100ng	D1	6,21
RiboG_sstotSmarter_100ng	D2	6,72
RiboZ_sstotSmarter_100ng	A1	4,18
RiboZ_sstotSmarter_100ng	A2	2,6
RiboZ_sstotSmarter_100ng	B1	1,96
RiboZ_sstotSmarter_100ng	B2	1,73
RiboZ_sstotSmarter_100ng	C1	2,83
RiboZ_sstotSmarter_100ng	C2	2,06
RiboZ_sstotSmarter_100ng	D1	2,38
RiboZ_sstotSmarter_100ng	D2	2,19
RiboZ_sstotSmarter_10ng	A1	5,72
RiboZ_sstotSmarter_10ng	A2	5,08
RiboZ_sstotSmarter_10ng	B1	3,65
RiboZ_sstotSmarter_10ng	B2	3
RiboZ_sstotSmarter_10ng	C1	2,1
RiboZ_sstotSmarter_10ng	C2	2,1
RiboZ_sstotSmarter_10ng	D1	2,34
RiboZ_sstotSmarter_10ng	D2	2,63
SmarterUL_NexteraXT_1ng_130pg	A1	4,22
SmarterUL_NexteraXT_1ng_130pg	A2	3,8
SmarterUL_NexteraXT_1ng_130pg	B1	4,21
SmarterUL_NexteraXT_1ng_130pg	B2	4,72
SmarterUL_NexteraXT_1ng_130pg	C1	3,95
SmarterUL_NexteraXT_1ng_130pg	C2	4,27
SmarterUL_NexteraXT_1ng_130pg	D1	5,23
SmarterUL_NexteraXT_1ng_130pg	D2	4,91
SmarterUL_NexteraXT_1ng_750pg	A1	4,17
SmarterUL_NexteraXT_1ng_750pg	A2	3,82
SmarterUL_NexteraXT_1ng_750pg	B1	3,75
SmarterUL_NexteraXT_1ng_750pg	B2	4,38
SmarterUL_NexteraXT_1ng_750pg	C1	3,87
SmarterUL_NexteraXT_1ng_750pg	C2	3,76
SmarterUL_NexteraXT_1ng_750pg	D1	4,24
SmarterUL_NexteraXT_1ng_750pg	D2	4,46
mRNATruseq_1ug	A1	1,58
mRNATruseq_1ug	A2	1,32
mRNATruseq_1ug	B1	2,64
mRNATruseq_1ug	B2	2,67
mRNATruseq_1ug	C1	1,59
mRNATruseq_1ug	C2	1,69
mRNATruseq_1ug	D1	2,4

mRNATruseq_1ug	D2	2,35
ssmRNATruseq_100ng	A1	2,68
ssmRNATruseq_100ng	A2	2,91
ssmRNATruseq_100ng	B1	4,38
ssmRNATruseq_100ng	B2	5,03
ssmRNATruseq_100ng	C1	3,51
ssmRNATruseq_100ng	C2	3,52
ssmRNATruseq_100ng	D1	3,95
ssmRNATruseq_100ng	D2	4,11
ssmRNATruseq_1ug	A1	1,13
ssmRNATruseq_1ug	A2	1,16
ssmRNATruseq_1ug	B1	2,77
ssmRNATruseq_1ug	B2	2,71
ssmRNATruseq_1ug	C1	1,85
ssmRNATruseq_1ug	C2	1,8
ssmRNATruseq_1ug	D1	2,67
ssmRNATruseq_1ug	D2	2,74
sstotRNATruseq_100ng	A1	5,34
sstotRNATruseq_100ng	A2	4,72
sstotRNATruseq_100ng	B1	3,22
sstotRNATruseq_100ng	B2	3,39
sstotRNATruseq_100ng	C1	8,17
sstotRNATruseq_100ng	C2	4,68
sstotRNATruseq_100ng	D1	4,62
sstotRNATruseq_100ng	D2	3,82
sstotRNATruseq_1ug	A1	3,54
sstotRNATruseq_1ug	A2	3,17
sstotRNATruseq_1ug	B1	1,75
sstotRNATruseq_1ug	B2	1,36
sstotRNATruseq_1ug	C1	2,03
sstotRNATruseq_1ug	C2	2,55
sstotRNATruseq_1ug	D1	2,48
sstotRNATruseq_1ug	D2	2,14

Supplementary Table S1: rRNA mapping percentages for all samples and conditions.

Condition	Threshold (CPM) 0.1	1	10
RiboG_sstotSmarter_100ng_2x2M	18182	18182	10985
RiboG_sstotSmarter_100ng_2x5M	22068	18669	11274
RiboG_sstotSmarter_100ng_2x10M	25202	19842	11556
RiboG_sstotSmarter_100ng_2x25M	29391	20041	11741
RiboZ_sstotSmarter_100ng_2x2M	18307	18307	11717
RiboZ_sstotSmarter_100ng_2x5M	21326	18791	12013
RiboZ_sstotSmarter_100ng_2x10M	23257	19703	12137
RiboZ_sstotSmarter_100ng_2x25M	25215	19680	12305
RiboZ_sstotSmarter_10ng_2x2M	20662	20662	13344
RiboZ_sstotSmarter_10ng_2x5M	23311	20698	13365
RiboZ_sstotSmarter_10ng_2x10M	25083	21399	13296
RiboZ_sstotSmarter_10ng_2x25M	26694	21240	13299
mRNASmarterUL_XT1ng_130pg_2x2M	19462	16877	11037
mRNASmarterUL_XT1ng_130pg_2x5M	22497	18433	11179
mRNASmarterUL_XT1ng_130pg_2x10M	24702	18407	11250
mRNASmarterUL_XT1ng_130pg_2x25M	25205	18655	11296
mRNASmarterUL_XT1ng_750pg_2x2M	19401	16882	10858
mRNASmarterUL_XT1ng_750pg_2x5M	23423	18409	11186
mRNASmarterUL_XT1ng_750pg_2x10M	24680	18425	11226
mRNASmarterUL_XT1ng_750pg_2x25M	25108	18764	11290
ssmRNATruseq_1ug_2x2M	19662	17235	11294
ssmRNATruseq_1ug_2x5M	22876	18324	11664
ssmRNATruseq_1ug_2x10M	25489	18602	11832
ssmRNATruseq_1ug_2x25M	26130	19083	11918
mRNATruseq_1ug_2x2M	20648	17805	11616
mRNATruseq_1ug_2x5M	24341	19590	11814
mRNATruseq_1ug_2x10M	27535	19628	11946
mRNATruseq_1ug_2x25M	28241	19766	12062
ssmRNATruseq_100ng_2x2M	19647	17157	10853
ssmRNATruseq_100ng_2x5M	22725	18262	11115
ssmRNATruseq_100ng_2x10M	25127	18533	11260
ssmRNATruseq_100ng_2x25M	25739	18773	11314
sstotRNATruseq_1ug_2x2M	20273	20273	12169
sstotRNATruseq_1ug_2x5M	24356	19714	12439
sstotRNATruseq_1ug_2x10M	27798	20196	12658
sstotRNATruseq_1ug_2x25M	29865	20548	12738
sstotRNATruseq_100ng_2x2M	20187	20187	12063
sstotRNATruseq_100ng_2x5M	24303	20765	12561
sstotRNATruseq_100ng_2x10M	27779	20705	12734
sstotRNATruseq_100ng_2x25M	32503	20985	12904

Supplementary Table S2: Number of detected genes for thresholds 0.1, 1 and 10 CPM.

Sample	RIN score
Human Universal Reference Total RNA	7,7
First Choice Human Brain Reference	7,3
A	7,8
B	7,4
C	7,7
D	7,7

Supplementary Table S3: BioAnalyzer RIN scores for the original samples as well as the samples A, B, C and D.

Kits used for sample preparation	Catalogue number
TruSeq® RNA Sample Prep Kit v2 – Set A (48 samples)	RS-122-2001
TruSeq® Stranded mRNA LT – Set A (48 samples, 12 indexes)	RS-122-2101
TruSeq® Stranded Total RNA LT (w/ Ribo-Zero™ Gold) – Set A (48 samples, 12 indexes)	RS-122-2301
SuperScript® II Reverse Transcriptase (4 x 10 000 units)	18064071
Ribo-Zero Magnetic Gold Kit (Human/Mouse/Rat) – 24 reactions	035MRZG12324<br>RZG1224
NucleoSpin® RNA XS (50 columns)	740902.50
RiboGone – mammalian (24 reactions)	634847
SMARTer Stranded RNA-Seq Kit (12 index, for Illumina)	634837
SMART-Seq v7 Ultra Low Input RNA Kit for Sequencing (24 reactions)	634889
NEXTERAR® XT DNA SAMPLE Prep Kit (24 SMP)	FC-131-1024
NEXTERA® XT DNA INDEX Kit (24 IDXS,96 SMP)	FC-131-1001

Supplementary Table S4: catalog numbers for the reagents (kits) used in this study.
